# Fracture resistance of resin based and lithium disilicate endocrowns. Which is better? – A systematic review of *in-vitro* studies

**DOI:** 10.1080/26415275.2021.1932510

**Published:** 2021-07-22

**Authors:** Joshna Beji Vijayakumar, Preethi Varadan, Lakshmi Balaji, Mathan Rajan, Rajeswari Kalaiselvam, Sindhu Saeralaathan, Arathi Ganesh

**Affiliations:** Department of Conservative Dentistry & Endodontics, Sri Ramachandra Faculty of Dental Sciences, SRIHER (DU), Chennai, India

**Keywords:** Ceramics, endocrown, hybrid composite, indirect resin, lithium disilicate, polymers

## Abstract

**Objectives:** The primary objective of this systematic review is to compare the fracture resistance of lithium disilicate (LDS)-based endocrowns and resin-based (RB) endocrowns of in-vitro studies, and the secondary objective is to compare their catastrophic failures.

**Materials and Methods:** The review protocol was registered in the P ROSP ERO database (CRD42020166201). A comprehensive literature search was done in PubMed, Cochrane, EBSCOhost and Google Scholar using key terms. Only in-vitro studies that compared fracture resistance of LDS-based endocrowns and indirect RB endocrowns in molars were included. Data extraction, risk of bias assessment and qualitative analysis of the included studies were performed.

**Results:** Five studies were included in this systematic review. The overall risk of bias for the included studies was moderate. Under axial loading, RB endocrowns showed similar fracture resistance when compared with LDS endocrowns. However, they showed better fracture resistance when compared with zirconia reinforced lithium silicate (ZLS) endocrowns. Furthermore, RB endocrowns showed fewer catastrophic failures than LDS-based endocrowns.

**Conclusions:** RB endocrowns have similar or better fracture resistance and fewer catastrophic failures when compared to LDS-based endocrowns.

## Introduction

Traditionally, post and core-retained crowns were the post-endodontic restoration of choice for teeth with extensive coronal loss. The post space preparation and the tooth preparation for full coverage crowns in this traditional approach cause further loss of tooth structure in the already mutilated teeth and can eventually lead to catastrophic fractures [1]. However, due to the advancement in the dental materials and adhesive technology, endocrowns are recommended as conservative post endodontic restorations for structurally compromised posterior teeth where there is greater than or equal to half the residual tooth structure present and provided the occlusion is favorable [2] and are also indicated even in endodontically treated teeth with extensive coronal loss with at least 3 mm intra-pulp chamber depth, 2 mm axial wall thickness and short clinical crown height [3].

Endocrowns are minimally invasive, residual tooth structure-oriented, cuspal coverage restorations that extend into the pulp chamber of the endodontically treated teeth. The material of choice for endocrowns plays a critical role in the biomechanical stress distribution and thereby influences the longevity of the endodontically treated teeth. Lithium disilicate-based ceramic (LDSB) is considered one of the best restorative materials due to its excellent optical properties, high fracture strength and adhesive property [3]. However, it has disadvantages of causing wear of opposing natural teeth and possibly catastrophic failures. Indirect resin-based (RB) endocrowns are emerging as an alternative to ceramic endocrowns due to their improved mechanical properties, such as modulus of elasticity (12.8 GPa) which is similar to dentin (18.6 GPa) [4]. They also possess stress-absorbing property which can be beneficial to individuals with weak periodontium [5]. Furthermore, indirect RB endocrowns cause less wear of opposing natural teeth than ceramics and can be repaired intraorally with composites [6]. Because of these aforementioned characteristics, RB materials have recently been considered for fabricating endocrowns.

Hence, the primary objective of the current systematic review was to answer the focused research question: ‘In endodontically treated human permanent molars (P), do resin-based endocrowns (I) show better fracture resistance (PO) and less catastrophic failures (SO) when compared to lithium disilicate-based endocrowns (C) in in-vitro studies (S)?’.

PICOS format

P – Extracted human permanent molar teeth

I – LDS-based endocrowns

C – Resin-based endocrowns

O – Primary Outcome: Fracture resistance

Secondary Outcome: Catastrophic failure

S – *In-vitro* studies

## Materials and methods

### Protocol and registration

This systematic review was reported according to the Preferred Reporting Items for Systematic Reviews and Meta-Analyses guidelines [7]. The protocol was registered in the PROSPERO database (CRD42020166201).

### Literature search strategy

In the regard of identifying articles for this systematic review, a comprehensive literature search was performed using the following databases: PubMed, EBSCOhost and Cochrane Central Register of Clinical trials and a comprehensive hand search was done in addition using Google Scholar to find out whether any other remaining article related to this review was available that had not appeared during the above databases search. Reference lists of previous systematic reviews and selected studies were also searched to identify potentially eligible studies. The literature search was done from its inception up to January 2020. The Boolean operators (OR, AND) were used in between to combine the keywords used for the search strategy.

#### Study selection

The included articles for this systematic review were obtained by literature search up to January 2020 after they met the inclusion and exclusion criteria mentioned in Table 1.

**Table 1. Eligibility criteria for study selection.** 

Inclusion criteria	Exclusion criteria
*In-vitro* studies published in English Studies where experiments were conducted in endodontically treated human permanent molars Studies that compared the fracture resistance of lithium disilicate-based endocrowns (LDSB) with indirect resin-based endocrowns (RB)	Case reports Literature reviews Letters to the editor Short commentaries *In vivo* studies *Ex vivo* studies Dissertations Animal studies Studies reported in languages other than English Studies where endocrowns were fabricated by direct or semi-direct method only.

### Data extraction

Two reviewers (BJ and PV) independently screened the title and abstract for potentially relevant research articles related to the mentioned research question, and the full-text articles were screened to determine if they met the inclusion criteria. Any disagreement in the selection of studies was resolved by consensus or by a third reviewer (AG). The missing or incomplete data from the selected studies were collected from their authors through electronic communication.

The data extraction was done in Microsoft Word in a standardized form that included author name, year of the study published, tooth type, sample size, groups evaluated, dimensions of tooth preparation, endocrown surface treatment, bonding technique, outcomes evaluated, aging simulation, load cell diameter, crosshead speed, loading direction and area, fracture resistance values and percentages of catastrophic failure.

### Quality assessment of included studies

The Quality Assessment of the included studies was performed using Review Manager 5.3 software. Parameters significant to this research question were identified from the checklist for reporting *in-vitro* studies (CRIS) guidelines [8], a previous systematic review [9] and were modified to include the following parameters:

- Teeth randomization
- Sample size calculation
- Teeth with similar morphology
- Information on sample preparation and handling
- Aging simulation
- Outcome data with a coefficient of variation lower than 50%.

Articles that reported only one or two of the above-mentioned parameters were considered to have ‘high risk of bias’, three or four parameters as ‘medium risk of bias’ and five or six parameters as ‘low risk of bias’.

## Results

### Study characteristics (included and excluded studies)

**Figure 1.**
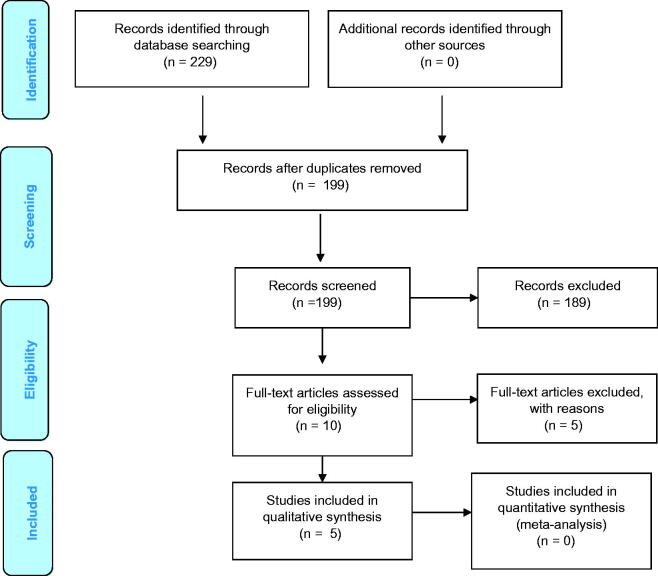
Flow diagram of study selection according to PRISMA statement.

The article selection process in the form of a flowchart is described in Figure 1. A total of 229 articles were identified through database search; 25 records from PubMed, 5 from Cochrane Library, 21 from EBSCOhost and 178 from Google Scholar. The detailed search strategy in PubMed is shown in Table 2. After duplicates were removed, 199 articles were screened by title and abstract. A total of 189 articles were excluded because they did not meet the eligibility criteria. The full text of the remaining 10 articles was assessed, and 5 articles [10–14] were excluded as they did not meet the eligibility criteria (Table 3). A total of five articles were included for qualitative analysis. Quantitative analysis was not performed due to the heterogenicity in the methodology.

**Table 2.** Search strategy used in PubMed.

((((((((((((‘endocrown*’[Title/Abstract]) OR ‘endo crown*’[Title/Abstract]) OR ‘endodontic crown*’[Title/Abstract]) OR ‘post free’[Title/Abstract]) OR ‘no post’[Title/Abstract]) OR ‘no buildup crown*’[Title/Abstract]) OR ‘no build up crown*’[Title/Abstract]) OR ‘depulped restoration*’[Title/Abstract])) AND ((‘molar*’[Title/Abstract]) OR ‘endodontically treated molar*’[Title/Abstract])) AND ((((((((((((‘lithium disilicate’[Title/Abstract]) OR ‘lithium di silicate*’[Title/Abstract]) OR ‘lithium silicate’[Title/Abstract]) OR ‘IPS emax’[Title/Abstract]) OR ‘CAD/CAM’[Title/Abstract]) OR ‘Computer aided design/computer aided manufacturing’[Title/Abstract]) OR ‘pressed ceramic*’[Title/Abstract]) OR ‘monolithic’[Title/Abstract]) OR ‘layered ceramic*’[Title/Abstract]) OR ‘emax CAD’[Title/Abstract]) OR ‘emax press’[Title/Abstract]) OR ‘glass ceramic*’[Title/Abstract]))AND (((((((((((((((((‘composite*’[Title/Abstract]) OR ‘resin*’[Title/Abstract]) OR ‘nanofill composite*’[Title/Abstract]) OR ‘Lava ultimate’[Title/Abstract]) OR ‘cerasmart’[Title/Abstract])) OR ‘resin nanoceramic*’[Title/Abstract]) OR ‘nano ceramic*’[Title/Abstract]) OR ‘polymer infiltrated ceramic network’[Title/Abstract]) OR ‘PICN’[Title/Abstract]) OR ‘polymer infiltrated ceramic*’[Title/Abstract]) OR ‘nanoceramic*’[Title/Abstract]) OR ‘ENAMIC’[Title/Abstract]) OR ‘Hybrid ceramic*’[Title/Abstract]) OR ‘resin interpenetrating matrix’[Title/Abstract]) OR ‘MZ100 block*’[Title/Abstract]) OR ‘paradigm MZ100 block*’[Title/Abstract])) AND (((((((((‘fracture’[Title/Abstract]) OR ‘fracture resistance’[Title/Abstract]) OR ‘fracture strength’[Title/Abstract]) OR ‘fracture toughness’[Title/Abstract]) OR ‘success rate’[Title/Abstract]) OR ‘survival rate’[Title/Abstract]) OR ‘failure’[Title/Abstract]) OR ‘failure rate’[Title/Abstract]) OR ‘fracture rate’[Title/Abstract])

**Table 3.** List of excluded studies.

S. no	Author	Year	Reason for exclusion
1	Skalskyi et al. [10]	2018	RB endocrowns were fabricated by semi-direct method.
2	Dartora et al. [11]	2019	The outcome assessed was fatigue performance
3	Sedrez-Porto et al. [12]	2019	RB endocrowns were fabricated by semi-direct method.
4	Altier et al. [14]	2018	RB endocrowns were fabricated semi-direct method
5	Sedrez-Porto et al. [13]	2020	RB endocrowns were fabricated by direct method.

### Characteristics of the included studies

The characteristics of the included studies are presented in Tables 4A and 4B. The included articles were published between 2015 and 2020. Both maxillary and mandibular molars were included, and a total of 172 LDSB and RB endocrowns were assessed. The number of LDSB endocrowns was 96, out of which LDS was 66, and zirconia reinforced lithium silicate (ZLS) was 30. The number of RB endocrowns in the included studies was 76, among which resin nanoceramic (RNC) were 60 and polymer infiltrated ceramic network (PICN) were 16.

**Table 4.** Characteristics of included studies.

A							
Author	Type of teeth	Sample size of study	Groups (endocrown)	Tooth preparation dimensions	Endocrown surface treatment	Bonding technique	Outcomes evaluated
El-Damanhoury et al. [15]	Maxillary Molar	30	Aluminosilicate- Feldspathic ceramic (CEREC Blocks) LDS (emax.CAD) RNC (LAVA ultimate)	Intra coronal depth: 2 mm Internal taper: 8–10◦ Wall thickness: 2 ± 0.2 mm	LDS: 5% HF; 60 s + silane RNC: Sandblasting + silane	3 step etch & rinse + Dual cure resin (Variolink II)	Fracture resistance Failure mode Microleakage
Gresnigt et al. [16]	Molars	60	LDS (IPS.emax CAD) RNC (Lava Ultimate)	Occlusal preparation: 1 mm above CEJ	LDS: 4.9% HF; 20 s + silane RNC: silica coated + silane	IDS – 3 step etch & rinse + IDS silica coated + enamel etched + Silane + Primer + adhesive + dual cure resin (Variolink II)	Fracture strength (axial & lateral) Failure type Weibull characteristic
El Ghoul et al. [17]	Mandibular molars	80	LDS (IPS emax) ZLS (Vita suprinity) RNC (Cerasmart) Control: LDS crown	Intra coronal depth: 4 mm Oclussal divergence: 8◦	LDS & ZLS: 5% HF; 20 s + silane RNC: 5% HF; 60 s + silane	2 step etch & rinse + dual cure resin (G-CEM)	Fracture resistance (axial & lateral) Failure mode
Taha et al. [18]	Mandibular first molar	40	LDS (emax CAD) PICN (Vita ENAMIC) ZLS (Celtra Duo) RNC (Cerasmart)	Occlusal reduction: 2 mm Cavity depth: 6 mm from central groove Divergence: 8˚	LDS: 4.5% HF; 20 s + silane PICN & RNC: 4.5% HF; 60 s + silane ZLS: 4.5% HF; 30 s + silane	Selective enamel etching + self-adhesive resin (Rely X Unicem2 Automix)	Fracture resistance Marginal gap
Ali and Moukarab [19]	Mandibular first molars	24	LDS (IPS e.max CAD) with DME LDS (IPS e.max CAD) without DME PICN (Vita ENAMIC) with DME PICN (Vita ENAMIC) without DME	Cervical margin: 2 mm above CEJ Cavity depth: 3 mm MMC : width-2 mm, depth-2 mm, 2 mm apical to CEJ	LDS: 9.5% HF; 90 sec + silane PICN: 9.5% HF; 60 sec + silane	2 step etch & rinse + Dual cure self-adhesive resin (Rely X Unicem clicker)	Marginal adaptation Fracture resistance

B						
Study	Aging simulation	Load cell diameter (mm)	Crosshead speed (mm/min)	Loading direction and area	Fracture strength (Newton)	Catastrophic failure (%)
El-Damanhoury et al. [15]	5000 thermocycles; 5 °C and 55 °C; 30 s each	2.5	0.5	Axial: incline of palatal cusp	RNC (1583.28 ± 170.55) LDS (1368.75 ± 237.34)	LDS-70 RNC-0
Gresnigt et al. [16]	10,000 thermocycles; 5 °C and 55 °C; 30 s each	–	–	Axial: central fissure Lateral: tooth – endocrown interface	Axial RNC (2675 ± 58.8) LDS (2428 ± 566) Lateral RNC (838 ± 169) LDS (1118 ± 173)	Axial LDS-30 RNC 30 Lateral LDS-50 RNC-20
El Ghoul et al. [17]	300,000 thermocycles; 5 °C and 55 °C; 30 s each Masticatory simulation: 5 mm vertical movement and 5 mm lateral movement; 50 N axial force; 1.6 Hz frequency; 4 mm stainless steel ball; 3,000,000 cycles	3	0.5	Axial: center of the occlusal surface Lateral: tooth-restoration interface	Axial LDS (2914 ± 205) ZLS (2279 ± 290) RNC (2752 ± 242) Lateral LDS (1516 ± 202) ZLS (1074 ± 153) RNC (1210 ± 97)	Axial LDS-50 RNC-40 ZLS- 40 Lateral LDS-60 RNC-20 ZLS-20
Taha et al. [18]	5000 thermocycles; 5 °C and 55 °C; 1 min each Masticatory simulation: 50 N vertical load; 6 × 10^5^ cycles	6	0.5	Axial: central fossa	RNC (1508.5 ± 421.7) PICN ( 1241.5 ± 249.8) ZLS (886.9 ± 197.7) LDS (1478.9 ± 412.2)	–
Ali and Moukarab [19]	10,000 thermocycles; 5 °C and 55 °C; 30 s each	6	0.5	Axial: over a tin foil sheet; middle of the occlusal surface	LDS with DME (1478.2 ± 107.5 ) LDS without DME (1217.2 ± 76.4) PICN with DME (1049 ± 66.3) PICN without DME (928. 7 ± 69.5)	–

LDS: lithium disilicate; RNC: resin nanoceramic; ZLS: zirconia reinforced lithium silicate; PICN: polymer infiltrated ceramic network; DME: deep marginal elevation; MMC: mesial marginal cavity; HF: hydrofluoric acid.

Under axial loading, the fracture resistance value of LDSB endocrowns ranged from 886.9 ± 197.7 to 2914 ± 205 N; LDS (IPS Emax from 1217.2 ± 76.4 N to 2914 ± 205 N); ZLS (Celtra Duo 886.9 ± 195.7 N and Vita suprinity 2279 ± 290 N). The fracture resistance values of RB endocrowns ranged from 928.7 ± 695 N to 2752 ± 242 N; RNC endocrowns from 1508.5 ± 421.7 N to 2752 ± 242 N; PICN endocrowns from 928.7 ± 69.5 N to 1241.5 ± 249.8 N [15–19]. Two studies [15,16] used Lava Ultimate (1583.28 ± 170.55 N and 2675 ± 58.8 N) and two studies [17,18] used Cerasmart (1508.5 ± 421.7 N and 2752 ± 242 N) for the fabrication of RNC endocrowns.

Under lateral loading, LDS endocrowns (1118 ± 173 and 1516 ± 202 N) showed higher fracture resistance than RNC endocrowns (838 ± 169 and 1210 ± 97 N) [16,17]. Both LDS and RNC showed lower fracture resistance under lateral loading than that under axial loading.

#### Quality assessment results

**Figure 2.**
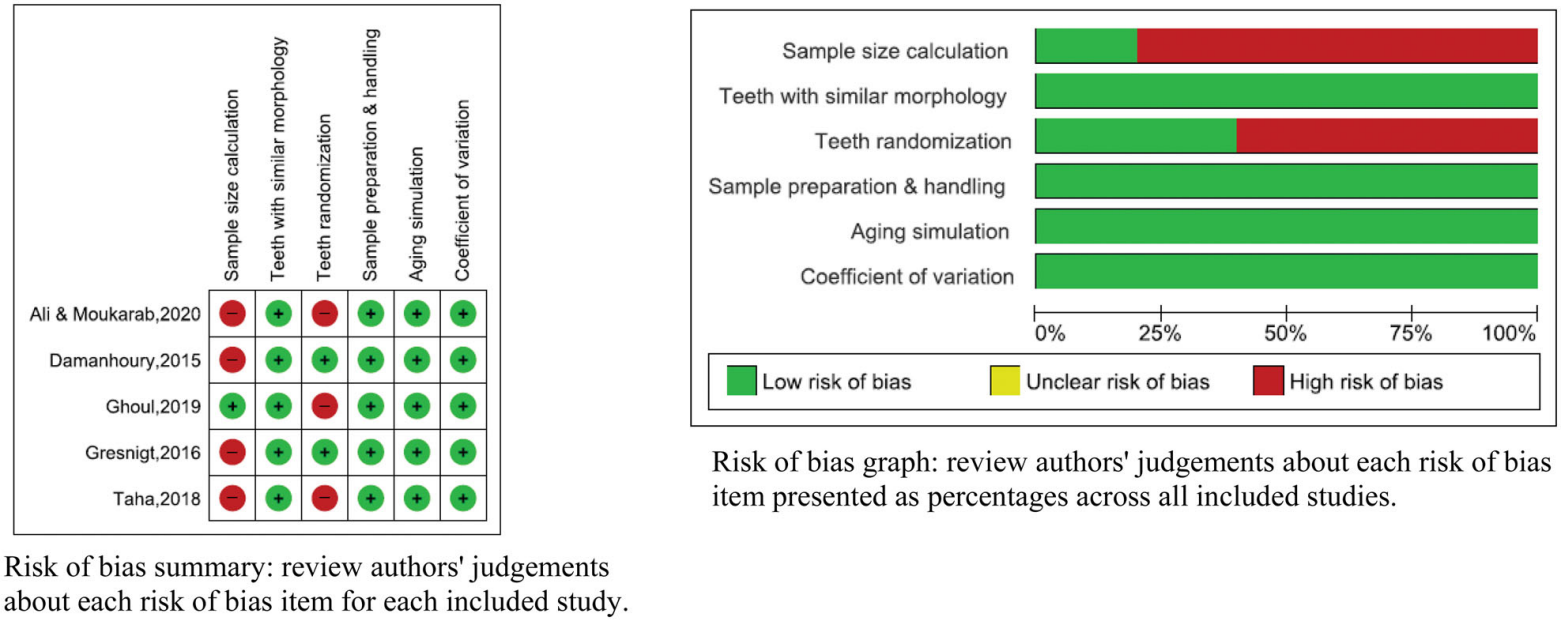
Risk of bias assessment.

The risk of bias assessment is given in Figure 2. Three studies [15–17] were assessed as having low risk of bias; two studies as having a moderate risk of bias; none of the studies was assessed as having a high risk of bias. The included studies did not report particularly on sample size calculation [15,16,18,19] or teeth randomization [17] or both [18,19].

## Discussion

Endodontically treated teeth are more susceptible to fracture than vital teeth because of the amount of tooth structure loss due to caries, access cavity preparation, canal preparation, aggressive irrigation solutions and intracanal medicaments [20,21]. Additionally, in teeth with extensive coronal loss, the placement of a post in order to retain the core requires further tooth structure removal that weakens the peri cervical dentin and predisposes to vertical root fracture [20]. Recently, endocrowns have been considered as alternatives to post and core retained crowns in molars as they are less susceptible to fracture than the latter [3,22–25]. The concept of endocrowns was first introduced by Pissis in 1995, and was known as the ‘monobloc porcelain technique’ [26]. The term ‘endocrown’ was first coined by Bindl and Mormann in 1999 [27]. Endocrowns are minimally invasive restorations and are useful in teeth having short clinical crowns with inadequate interocclusal clearance, curved roots, slender roots or calcified root canals. The mechanical properties of restorative material play an essential role in the success of the post-endodontic restorations and the survival of the endodontically treated teeth [16,17]. Hence, this systematic review compared the fracture resistance of LDSB endocrowns and RB endocrowns in molars.

In the included studies, the LDSB endocrowns assessed were LDS and ZLS and the RB endocrowns assessed were RNC and PICN. All endocrowns in the included studies were fabricated by CAD/CAM milling technique.

### Lithium disilicate (LDS) vs. resin nanoceramic (RNC)

Under axial loading, three studies. [16–18]) showed no statistically significant difference between LDS and RNC endocrowns. El Damanhoury et al. [15] showed that RNC had significantly better fracture resistance than LDS endocrowns. The high fracture strength of RNC endocrowns is attributed to the unique composition. RNC (Lava Ultimate) consists of 80% nanoceramic particles and 20% resin matrix [15]. RNC (Cerasmart) blocks are fabricated under high temperature and pressure to achieve high volume fraction filler and high conversion rate (85%), which enhances their fracture resistance [18].

Under lateral loading, two studies [16,17] reported that LDS endocrowns showed better fracture resistance than RNC endocrowns, which is attributed to their excellent micromechanical interlocking with the resin cement and their adhesion between the tooth surface and resin cement [17].

### Lithium disilicate (LDS) vs. polymer infiltrated ceramic network (PICN)

Under axial loading, Taha et al. [18] showed no significant difference between LDS and PICN endocrowns. However, a study by Ali and Moukarab [19] found that LDS endocrowns had significantly better fracture resistance than PICN endocrowns. The fracture strength of LDS (IPS e.max) is attributed to the high crystal filler volume (70% volume of LDS), and to the filler particles being distributed in a tight interlocking fashion, counteracting the crack propagation [18,19]. PICN consists of a 75% volume ceramic network with a 25% volume polymer network. The polymer phase within the ceramic phase in PICN increases crack resistance and improves fracture resistance [18]. PICN has a modulus of elasticity similar to that of dentin, and hence it absorbs stress [19].

### Zirconia reinforced lithium silicate (ZLS) vs. resin nanoceramic (RNC)

Under axial loading, two studies [17,18]) showed that RNC endocrowns had significantly higher fracture resistance than ZLS endocrowns. This result could be attributed to the fact that ZLS has a low crystal filler volume (36% volume of LDS) than RNC with 80% nanoceramic filler volume [18,28].

Under lateral loading, one study reported that there was no statistically significant difference between ZLS and RNC endocrowns [17].

### Zirconia reinforced lithium silicate (ZLS) vs. polymer infiltrated ceramic network (PICN)

Only one study by Taha et al. [18] compared fracture resistance of ZLS and PICN endocrowns and revealed that ZLS endocrowns had significantly higher fracture resistance than PICN endocrowns under axial loading.

The aging simulation carried out before the fracture test influences the fracture resistance of the materials [29]. Accordingly, the fracture resistance values of endocrowns in the included studies were found to be indirectly proportional to the number of thermocycles. The study by El Ghoul et al. [17], which performed the least number of thermocycles (3000 cycles), reported the highest fracture resistance values. At the same time, the study by Ali and Moukarab [19], which performed the highest number of thermocycles (10,000 cycles), reported the least fracture resistance values.

Catastrophic failures under axial loading were assessed in three studies [15–17]; LDS endocrowns showed higher percentages of catastrophic fractures (30–70%) than RNC endocrowns (0–40%). Two studies [16,17] evaluated catastrophic failures under lateral loading; LDS endocrowns (50–60%) showed a higher number of catastrophic failures when compared to RNC endocrowns (20%). This could be because LDS is rigid and has a higher modulus of elasticity (100 GPa) than dentin and produce high-stress concentration at critical areas leading to catastrophic failures [14,15,30]. Whereas RNC has a low modulus of elasticity (12.8 GPa) close to that of dentin (18.6 GPa) [31,32], and it absorbs the stresses and distributes them more evenly [14].

The included studies showed heterogeneity in the reporting of tooth preparation dimensions in relation to the anatomical landmarks. The intra coronal depth and volume of the material influences the stress distribution pattern, which in turn influences the fracture mode of the restoration [32,33]. Therefore, future studies on endocrowns must consider reporting the dimensions of the intra-coronal depth of endocrowns, occlusal reduction, axial wall height from CEJ, the width of cervical sidewalk and degree of internal wall divergence. Also, future *in-vitro* studies must reduce the risk of bias, especially on sample size calculation and teeth randomization. The moderate quality of the included studies and the descriptive method of analysis in itself could be the limitations of the current systematic review which must therefore be interpreted with caution. Furthermore, high-quality randomized clinical trials are needed to support the inference of this review.

## Conclusion

Within the limitations of the current review, it can be concluded with a moderate level of evidence that under axial forces, RB endocrowns may have similar or even higher fracture resistance when compared with LDSB endocrowns. Furthermore, RB endocrowns tended to show fewer catastrophic failures when compared with LDSB endocrowns, which is of clinical significance.
